# Low-intensity pulsed ultrasound promotes chondrogenesis of mesenchymal stem cells via regulation of autophagy

**DOI:** 10.1186/s13287-019-1142-z

**Published:** 2019-01-22

**Authors:** Xiaoju Wang, Qiang Lin, Tingting Zhang, Xinwei Wang, Kai Cheng, Mingxia Gao, Peng Xia, Xueping Li

**Affiliations:** Department of Rehabilitation Medicine, Nanjing First Hospital, Nanjing Medical University, Nanjing, 210006 China

**Keywords:** Low-intensity pulsed ultrasound (LIPUS), Mesenchymal stem cells (MSCs), Chondrogenesis, Autophagy

## Abstract

**Background:**

Low-intensity pulsed ultrasound (LIPUS) can induce mesenchymal stem cell (MSC) differentiation, although the mechanism of its potential effects on chondrogenic differentiation is unknown. Since autophagy is known to regulate the differentiation of MSCs, the aim of our study was to determine whether LIPUS induced chondrogenesis via autophagy regulation.

**Methods:**

MSCs were isolated from the rat bone marrow, cultured in either standard or chondrogenic medium, and stimulated with 3 MHz of LIPUS given in 20% on–off cycles, with or without prior addition of an autophagy inhibitor or agonist. Chondrogenesis was evaluated on the basis of aggrecan (AGG) organization and the amount of type II collagen (COL2) and the mRNA expression of AGG, COL2, and SRY-related high mobility group-box gene 9 (SOX9) genes.

**Results:**

LIPUS promoted the chondrogenic differentiation of MSCs, as shown by the changes in the extracellular matrix (ECM) proteins and upregulation of chondrogenic genes, and these effects were respectively augmented and inhibited by the autophagy inhibitor and agonist.

**Conclusions:**

Taken together, these results indicate that LIPUS promotes MSC chondrogenesis by inhibiting autophagy.

## Background

Articular cartilage injury is a common complication of joint diseases like osteoarthritis. Due to its low innervation, poor blood supply, and low chondrocyte proliferation and migration, autologous cartilage repair capacity is very limited and can lead to irreversible joint dysfunction after injury [1].

The current therapeutic strategies of alleviating articular cartilage injury have unsatisfactory clinical outcomes. Although cartilage engineering can be a promising option [2], the poor regenerative capacity of chondrocytes precludes their use as the seeding cells [3].

Mesenchymal stem cells (MSCs) are highly proliferative self-renewing cells with multi-lineage differentiation ability and have become the most promising cell source for cartilage regeneration. In fact, autologous MSCs implanted into regions of defective cartilage differentiated into chondrocytes, indicating their potential for efficient cartilage repair [4].

However, MSCs have an inherently limited capacity of chondrogenesis, which in turn limits the therapeutic efficacy and outcome of MSC transplantation [3, 5–7]. Differentiation of MSCs into chondrocytes is influenced by the extracellular microenvironment, as well as several growth factors, primarily the transforming growth factor (TGF) [8]. However, little is known regarding the regulatory mechanisms of chondrogenesis.

Autophagy is a catabolic process that enables cells to recycle damaged proteins and organelles to ensure their survival during stress conditions [9, 10]. The autophagy-related genes such as Beclin1 and LC3 are essential for autophagosome formation in the MSCs [11, 12]. Studies show that autophagy plays an important role in MSC differentiation and regulates its therapeutic effects in inflammatory diseases [13, 14]. However, it is unclear whether autophagy also affects chondrogenesis of MSCs.

In recent years, studies have shown that mechanical stimulation affects the differentiation of MSCs [15, 16]. Low-intensity pulsed ultrasound (LIPUS) provides mechanical stimulation in the form of acoustic waves and has been used as an adjuvant physical therapy to treat musculoskeletal injuries. Recent studies show that LIPUS promotes cartilage repair, and stimulation of chondrocytes with LIPUS increases production of extracellular matrix (ECM) proteins like type II collagen (COL2) and aggrecan (AGG) [17–19]. In addition, LIPUS has been shown to facilitate TGF-β-mediated chondrogenesis of MSCs in vitro [20, 21]. These findings strongly indicate the potential of LIPUS in regenerating damaged cartilage via MSCs.

In this study therefore, we analyzed the effects of autophagy regulation and LIPUS on MSC chondrogenesis and found that LIPUS drives the chondrogenic differentiation of MSCs by inhibiting autophagy.

## Material and methods

### MSC isolation and culture

Bone marrow-derived MSCs (BMSCs) were isolated from 18 8-week-old male Sprague-Dawley (SD) rats as previously described [22, 23]. The experimental protocol was in accordance with the US National Institutes of Health’s Guidelines of Laboratory Animal Use and approved by the Nanjing Medical University Ethics Committee of Nanjing Hospital. Briefly, the bone marrow was flushed out from the femur cavity with low-glucose Dulbecco’s modified Eagle’s medium (DMEM; KeyGEN, Nanjing, Jiangsu, China) containing 10% fetal bovine serum (FBS; KeyGEN). After centrifuging the BM cell suspension for 10 min at 1000 rpm, the fat and other debris were removed and the remaining cells were washed twice with PBS. The cells were re-suspended in DMEM and cultured in petri dishes at 37 °C under 5% CO_2_. After reaching 80–90% confluency, the cells were trypsinized and re-seeded at the density of 2 × 10^6^ cells per dish. The MSCs were identified morphologically under a light microscope [13].

### Immuno-phenotypic characterization of MSC

The MSCs were obtained from each rat and identified respectively. The primary MSCs were harvested using 0.25% trypsin (KeyGEN), washed twice in PBS, and centrifuged at 400*g* for 5 min at room temperature. After re-suspending the cells in the staining buffer at the density of 2 × 10^6^/ml, 100-μl aliquots were incubated with FITC-conjugated rabbit anti-mouse CD90 and CD31 (Abcam, Cambridge, MA, USA) and unconjugated anti-CD44 and anti-CD45 (Santa Cruz, Dallas, TX, USA) for 15 min at 4 °C. The cells were washed once with ice-cold staining buffer and re-suspended in the buffer containing FITC-conjugated goat anti-rabbit IgG (Jackson, Philadelphia, Pennsylvania, USA) for 15 min at 4 °C. After washing again with ice-cold PBS containing 2% bovine serum albumin (BSA), the cells were acquired using a flow cytometer (FACS Calibur, BD Biosciences, SanJose, CA, USA). FITC-conjugated mouse IgG1 (R&D systems Inc., Minneapolis, MN, USA) was used as the isotype control for CD90 and CD31, and rabbit polyclonal IgG (Epitomics, Burlingame, CA, USA) for CD44 and CD45. The acquired cells were analyzed using WinMDI 2.8 software (The Scripps Institute, West Lafayette, IN, USA).

### Induction of chondrogenic differentiation

The MSCs were differentiated to chondrocytes in a three-dimensional pellet culture system as previously described [20, 24]. Briefly, the second generation MSCs were harvested (approximately 2 × 10^6^ cells) and pelleted by centrifuging at 300*g* for 5 min. The undisturbed pellet was cultured in chondrogenic medium (KeyGEN)—DMEM containing 10% FBS, 50 units/mL penicillin, 50 mg/mL streptomycin, 0.1 μM hexadecadrol, 0.1 mM Vitamin C, 50 μg/mL ascorbate 2-phosphate, 0.35 mM proline, 1 mM pyruvate, 10 ng/ml TGF-β3, 50 mg/mL ITS Premix, 6.25 μg/mL insulin, 6.25 μg/mL transferrin, 6.25 μg/mL sodium selenate, and 5.35 μg/mL linoleic acid—at 37 °C under 5% CO_2_. Control MSC pellets were re-suspended in basic medium (DMEM with 10% FBS). The culture medium was changed every 3 days until the pellets were harvested. The MSCs were cultured in chondrogenic medium for 10 days before analyses.

### LIPUS stimulation and autophagy agonist and inhibitor treatment

The tubes containing the differentiated MSCs were placed on the transducer (HT2009-1, Ito Corporation, Tokyo, Japan), and LIPUS waves of varying intensities (20 mW/cm^2^, 30 mW/cm^2^, 40 mW/cm^2^, or 50 mW/cm^2^) were transmitted through the bottom of the tube coated with a coupling agent as previously described [20]. The cells were treated once a day for 10 days at the on–off ratio of 20%, and irradiated with 3 MHz for 20 min in a humidified 37 °C incubator with 5% CO_2_. To determine the role of autophagy on the chondrogenic effects of LIPUS, the cells were incubated with the autophagy inhibitor 3-methyladenine (3-MA; Selleck, Houston, TX, USA) or agonist rapamycin (Selleck) before the LIPUS stimulation. During the LIPUS stimulation and the autophagy agonist and inhibitor treatment, the medium were changed every 3 days. When the medium were changed, the autophagy agonist and inhibitor were re-added to the medium.

### Western blotting

Protein was extracted from the cells using a total protein extraction kit (KeyGEN), and equal amounts of protein (20–25 μg) per sample were loaded into sodium-dodecyl sulfate polyacrylamide (SDS-PA) gels and resolved by electrophoresis. After blotting the proteins onto nitrocellulose membranes, the latter were blocked with skim milk for 2 h at room temperature and incubated overnight with anti-Beclin1 (1:1000; Cell Signaling Technology, Danvers, MA, USA), anti-LC3 (1:1500; Novus Biological, Littleton, Colorado, USA), and anti-β-actin (1:1000; Cell Signaling Technology) antibodies at 4 °C. The following day, the blots were washed thrice with Tween-20 in PBS and incubated with peroxidase-conjugated goat anti-mouse secondary antibody (1:5000; Santa Cruz, Dallas, TX, USA) at 37 °C for 2 h. After the final three washes, the membranes were developed by exposure to chemiluminescence reagents (ECL kit; KeyGEN).

### Electron microscopy

Harvested cells were washed in ice-cold PBS, fixed with 2% glutaraldehyde (Sigma-Aldrich, St. Louis, MO, USA), and washed twice with PBS. Cells were post-fixed with 1% osmium tetroxide (Sigma-Aldrich), dehydrated, and treated with propylene oxide (Sigma-Aldrich) before being embedded in epoxy resin (Sigma-Aldrich). The blocks were cut into thin sections, stained with lead citrate (Sigma-Aldrich), and observed under the electron microscope (JEM-1011, JEOL, Akishima, Tokyo, Japan).

### Immunofluorescence

The MSCs were seeded onto slides and cultured for 10 days. And then the MSCs were fixed with 4% paraformaldehyde for 30 min on ice, washed twice with PBS, and incubated with 3% H_2_O_2_-methanol solution at room temperature for 10 min. Micromass pellets were washed twice with PBS, fixed for 24 h in 10% formalin, embedded in paraffin, and cut into 5-μm thick sections. The latter were deparaffinized, rehydrated, and then washed with PBS. After a 5-min incubation with 0.5% Triton X-100 (KeyGEN), the cells/sections were blocked with 10% goat serum in PBS for 30 min and incubated overnight with anti-LC3B antibody (1:200; Novus Biological) at 4 °C. The slides were washed thrice with the blocking solution, incubated with fluorochrome-conjugated secondary antibody, and counterstained with diamidine phenylindole (DAPI; Molecular Probes, Waltham, MA, USA). The LC3 punctae were observed and counted under a confocal microscope (Dmi 6000-B, Leica, Brunswick, Germany) [25, 26].

### Immunocytochemistry (ICC)

The pellets were washed twice with PBS, fixed for 24 h in 10% formalin, embedded in paraffin, and cut into 5-μm thick sections. The pellet sections were incubated with 3% H_2_O_2_ in methanol at room temperature for 10 min to quench the endogenous peroxidase, washed thrice with PBS, and blocked with goat serum at room temperature for 20 min. The sections were then incubated overnight with anti-COL2 antibody (1:200; Acris, Herford, NRW, Germany) at 4 °C, washed thrice with PBS, and incubated with horseradish peroxidase (HRP)-conjugated anti rabbit secondary antibody (50 μl; Santa Cruz) at 37 °C for 30 min. After washing thrice with PBS, 3,3-diaminobenzidine (DAB) was added for color development, and the sections were counterstained with hematoxylin (KeyGEN). Three slides were observed per condition, and positively stained cells were counted in three randomly selected areas per slide.

### Toluidine blue staining

To determine the presence of glycosaminoglycans, the pellet sections were washed thrice with PBS, fixed with 4% paraformaldehyde at room temperature for 20 min, and washed again. The slides were then stained with toluidine blue for 30 min, washed with PBS, and observed under an inverted microscope.

### Quantitative real-time (qRT)-PCR

Total RNA was extracted from the micromass pellets using TRIzol reagent (Invitrogen, Waltham, MA, USA) according to the manufacturer’s instructions, and 1 μg per sample was reverse-transcribed using a PrimeScript™ RT reagent Kit with gDNA Eraser (Takara Bio, Inc., Otsu, Shiga, Japan). The PCR primers (Table 1) targeting glyceraldehyde 3-phosphate dehydrogenase (GAPDH), COL2, AGG, and SRY-related high mobility group-box gene 9 (SOX9) were designed based on cDNA sequences from the NCBI Sequence database using Primer Express® software, and primer specificity was confirmed using BLASTN search. The qRT-PCR was performed using SYBR® Green PCR Mix (Takara Bio, Inc.) on the ABI Prism 7500 Fast Real-Time PCR System (Applied Biosystems, Foster City, CA, USA). To quantify the relative expression of each chondrogenic gene, Ct values were normalized against the endogenous reference (ΔCt = Ct_target_ − Ct_GAPDH_) and compared with a calibrator using the 2^−ΔΔCt^ method (ΔΔCt = ΔCt_sample_ − ΔCt_calibrator_).

**Table 1:** Primer sequences for qRT-PCR

Gene	Primer sequences
GAPDH	Forward:5′-GGGAAACCCATCACCATCTT-3′
	Reverse:5′-CCAGTAGACTCCACGACATACT-3′
COL2	Forward:5′-CAAGGAGAAGCTGGACAGAAA-3′
	Reverse:5′-CTTAGGACCAGTCACTCCAGTA-3′
AGG	Forward:5′-TGAAGGCAACTCTCGTCTTATT-3′
	Reverse:5′-GTCAGGGTCGTAAGGGATTATG-3′
SOX9	Forward:5′-GACGTGCAAGCTGGGAAAGT-3′
	Reverse:5′-CGGCAGGTATTGGTCAAACTC-3′

*GAPDH* glyceraldehyde 3-phosphate dehydrogenase, *COL2* type II collagen, *AGG* aggrecan, *SOX9* sex-determining region Y-box 9

### Statistical analysis

All data were expressed as mean ± standard deviation (SD) of three independent experiments and analyzed using SPSS 23.0 software (IBM Corp, Armonk, NY, USA). Multiple groups were compared by single-factor analysis of variance (ANOVA) and two groups by pair-wise Student’s *t* test. *P* values < 0.05 were considered statistically significant.

## Results

### Characterization of MSCs

**Fig. 1:**
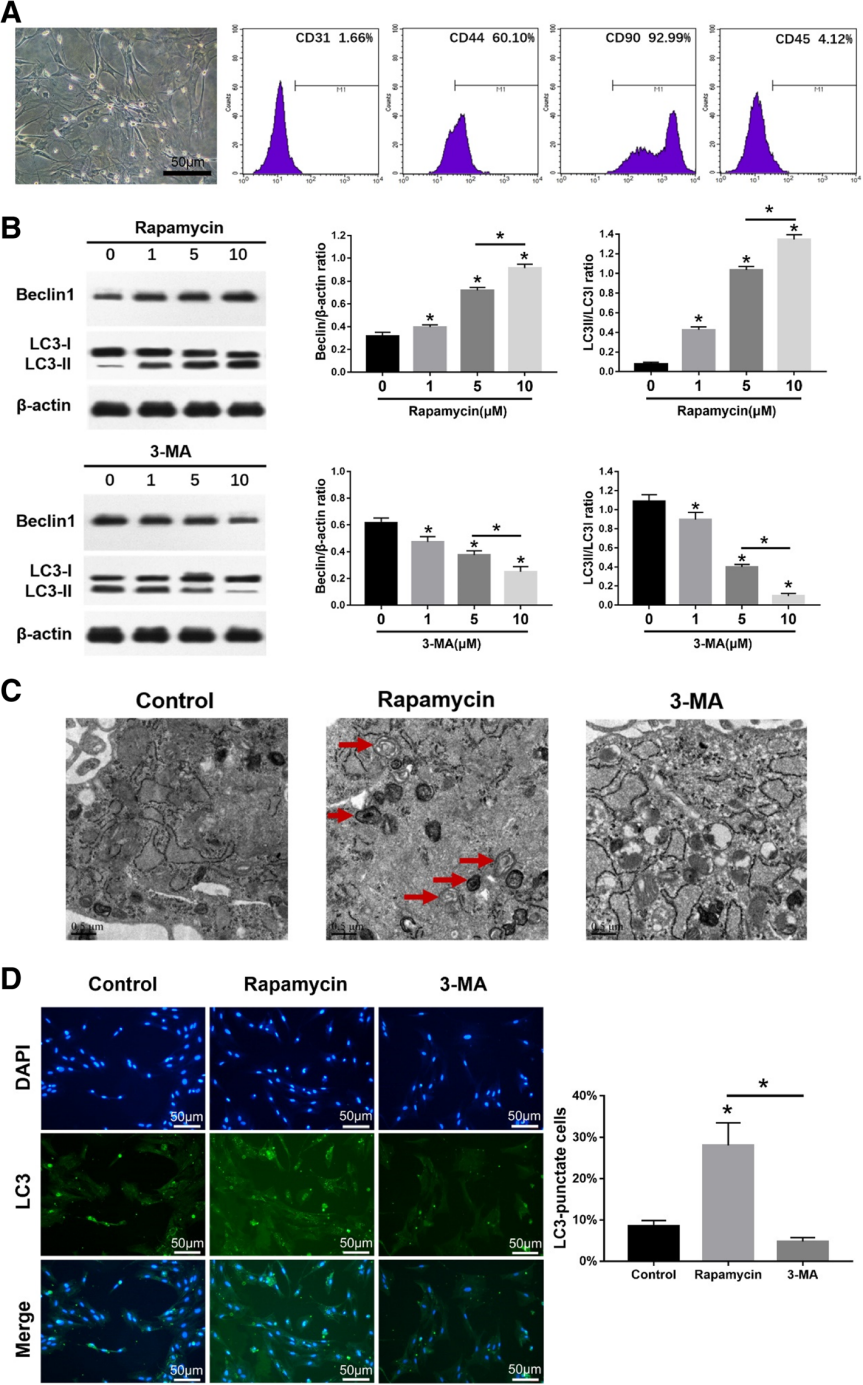
Characterization of MSCs and the effects of autophagy agonist and inhibitor. The MSCs were cultured in basic medium for 10 days before analyses. **a** Representative image showing second generation MSCs on day 10 of culture; scale bars = 50 μm. Flow cytometry histograms showing percentage of MSC marker-positive cells (red curve); purple shaded area represents the control. **b** Immunoblots showing the levels of Beclin1, LC3I, and LC3II in MSCs, with β-actin as the loading control. **c** Representative electron microscopy images showing autophagosomes (arrows); scale bars = 0.5 μm. **d** Left panel—representative immunofluorescence images showing LC3+ cells (green); scale bars = 50 μm. Right panel—bar graph comparing the number of LC3+ cells. The values are the mean ± SD of triplicate experiments; *P < 0.05

The MSCs appeared fusiform or triangular on day 10 of culture (Fig. 1a). Immuno-phenotyping showed that the MSCs were positive for CD44 (60.10 ± 3.73%) and CD90 (92.99 ± 7.33%) and negative for CD31 (1.66 ± 0.83%) and CD45 (4.12 ± 0.88%) (Fig. 1a).

### Effects of autophagy agonist and inhibitor on MSCs

The protein expression of autophagy-related gene Beclin1 and LC3 in MSCs was examined by western-blot analysis after rapamycin or 3-MA treatment for 24 h. Beclin1 expression and ratio of LC3II/LC3I in MSCs significantly increased (*p* < 0.05) after treatment with 1, 5, or 10 μM rapamycin, with the highest at 10 μM rapamycin (*p* < 0.05). Beclin1 expression and ratio of LC3II/LC3I in MSCs significantly decreased (*p* < 0.05) after treatment with 1, 5, or 10 μM 3-MA, with the lowest at 10 μM 3-MA (*p* < 0.05) (Fig. 1b). The autophagosome formation in MSCs was observed by electron microscopy. The morphometric ultrastructural analyses showed that autophagosomes were increased in rapamycin treatment group compared with the control group and 3-MA treatment group in MSCs (Fig. 1c). Immunofluorescence staining also showed that LC3-positive cells were significantly increased (*p* < 0.05) in rapamycin treatment group compared with the control group and 3-MA treatment group in MSCs (Fig. 1d).

### LIPUS inhibits autophagy and promotes chondrogenesis of MSCs

**Fig. 2:**
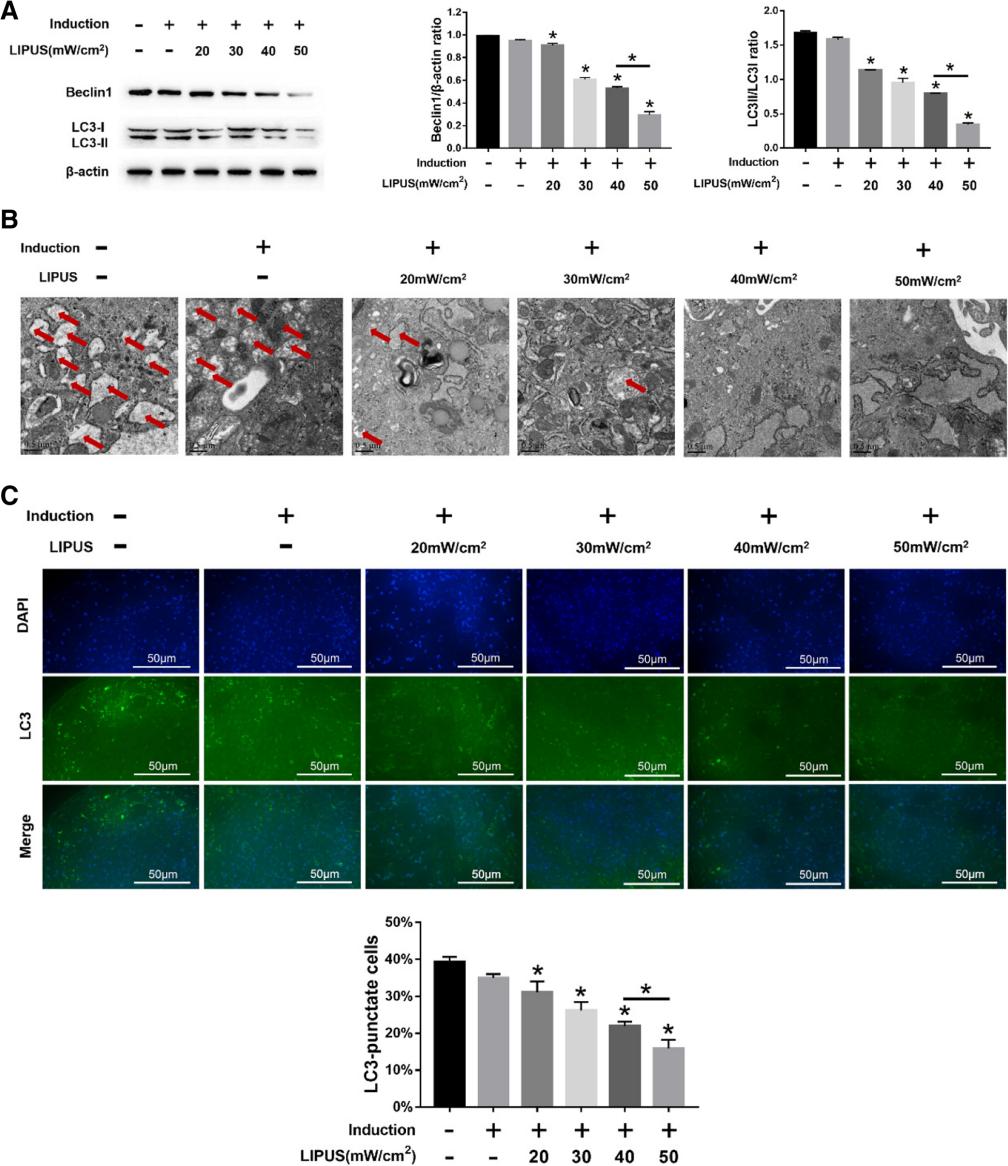
Effects of LIPUS on autophagy in MSCs. The MSCs were cultured in basic medium or chondrogenic medium for 10 days before analyses. **a** Immunoblots showing the levels of Beclin1, LC3I, and LC3II in differentiating MSCs stimulated with varying intensities of LIPUS, with β-actin as the loading control. **b** Representative electron microscopy images showing autophagosomes (arrows); scale bars = 0.5 μm. **c** Left panel—representative immunofluorescence images showing LC3+ cells (green); scale bars = 50 μm. Right panel—bar graph comparing the number of LC3+ cells. The values are the mean ± SD of triplicate experiments; *P < 0.05

**Fig. 3:**
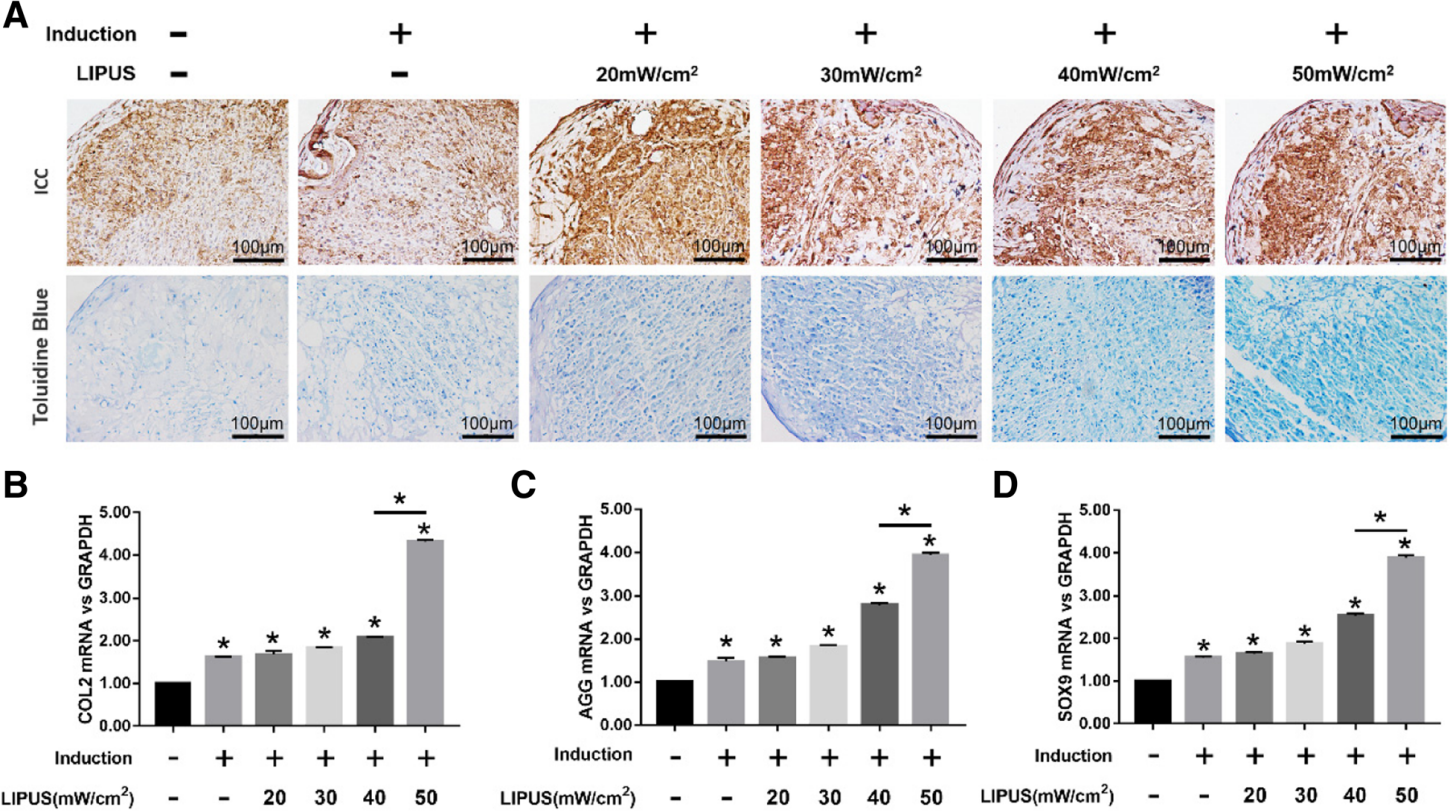
Effects of LIPUS on the chondrogenesis of MSCs. The MSCs were cultured in basic medium or chondrogenic medium for 10 days before analyses. **a** Representative images of ICC showing COL2+ cells (upper panel), and toluidine blue-staining showing AGG (lower panel) in differentiating MSCs stimulated with varying intensities of LIPUS; scale bars = 100 μm. **b**–**d** Bar graphs showing relative levels of COL2 (**b**), AGG (**c**), and SOX9 (**d**) mRNA in LIPUS-stimulated and unstimulated MSCs. The values are the mean ± SD of triplicate experiments; *P < 0.05

Stimulation with different intensities of LIPUS significantly decreased Beclin1 expression and LC3II/LC3I ratio in the MSC pellets (*p* < 0.05; Fig. 2a). Ultrastructural examination showed a significant decrease in the number of autophagosomes and LC3+ cells in MSCs undergoing chondrogenic differentiation following LIPUS stimulation (*p* < 0.05; Fig. 2b and c). The maximum anti-autophagic effect was seen at the intensity of 50 mW/cm^2^ intensity. To determine the effect of LIPUS on chondrogenesis, the ECM and chondrogenic markers were analyzed. A significantly higher number of COL2+ cells were seen in the differentiating MSCs stimulated with LIPUS (Fig. 3a), in addition to a greater density of AGG (Fig. 3a), compared to the unstimulated MSCs. Consistent with this, the COL2, AGG, and SOX9 genes were also upregulated (*p* < 0.05) following LIPUS stimulation (Fig. 3b-d). As with autophagy inhibition, the maximum pro-chondrogenic effect was seen at the intensity of 50 mW/cm^2^.

### Autophagy inhibits MSC chondrogenesis

**Fig. 4:**
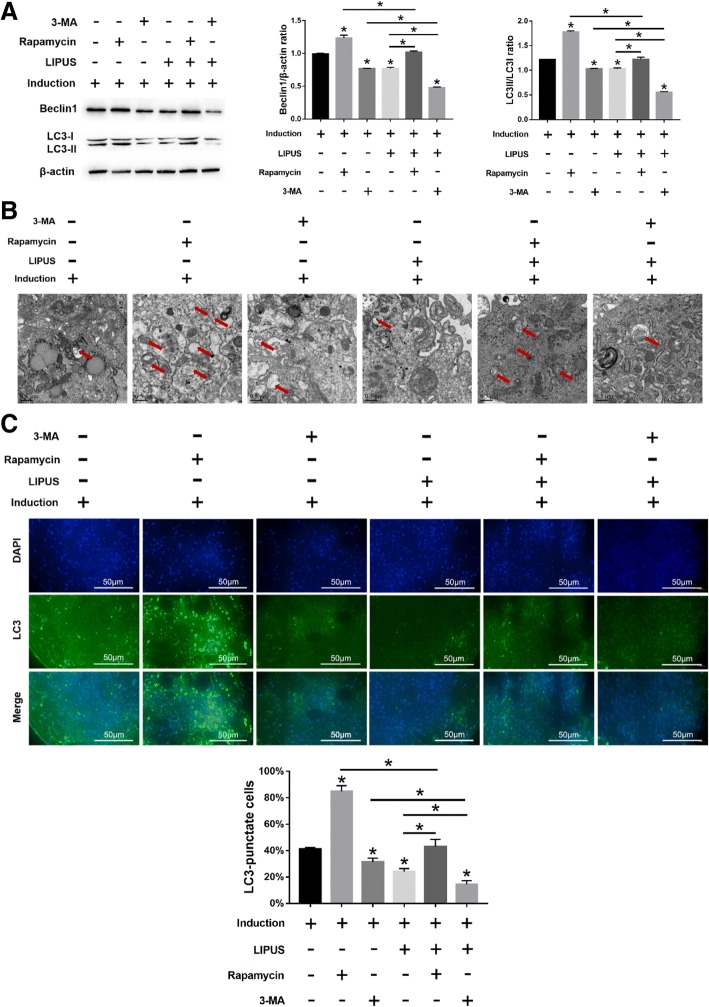
Effects of LIPUS on autophagy in MSCs treated with autophagy agonist or inhibitor. The MSCs were cultured in chondrogenic medium for 10 days before analyses. **a** Immunoblots showing the levels of Beclin1, LC3I, and LC3II in differentiating MSCs stimulated with varying intensities of LIPUS, with β-actin as the loading control. **b** Representative electron microscopy images showing autophagosomes (arrows); scale bars = 0.5 μm. **c** Left panel—representative immunofluorescence images showing LC3+ cells (green); scale bars = 50 μm. Right panel—bar graph comparing the number of LC3+ cells. The values are the mean ± SD of triplicate experiments; *P < 0.05

**Fig. 5:**
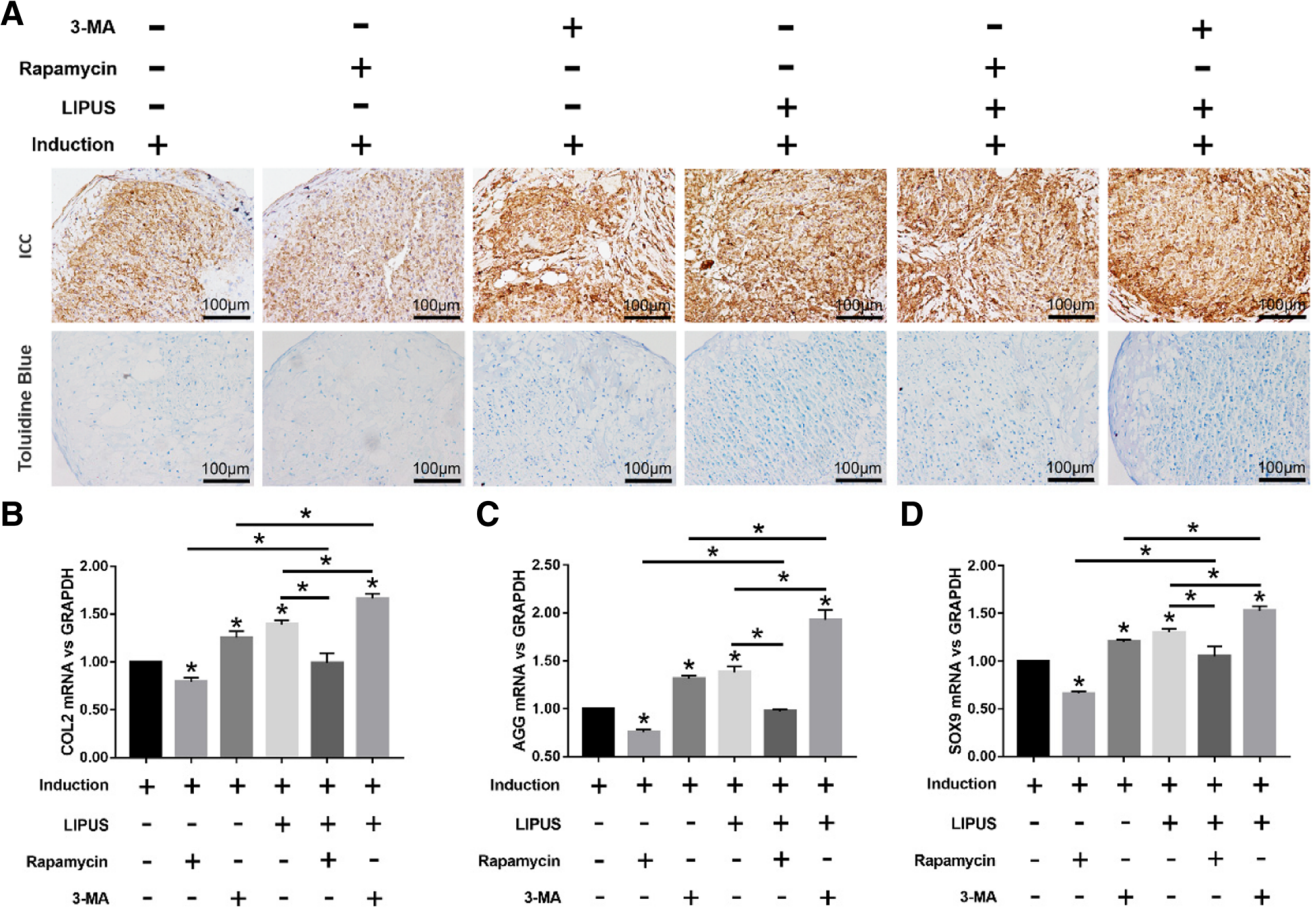
Effects of LIPUS on the chondrogenesis of MSCs treated with autophagy agonist or inhibitor. **a** Representative images of immunocytochemistry staining of COL2 (upper panel) and toluidine blue staining (lower panel) in differentiated MSCs stimulated with varying intensities of LIPUS; scale bars = 100 μm. **b**–**d** Bar graphs showing relative levels of COL2 (**b**), AGG (**c**), and SOX9 (**d**) mRNA in LIPUS-stimulated and unstimulated MSCs. The values are the mean ± SD of triplicate experiments; *P < 0.05

MSCs undergoing chondrogenic differentiation showed elevated Beclin1 expression and LC3II/LC3I ratio after rapamycin treatment (*p* < 0.05), which decreased significantly when treated with 3-MA (*p* < 0.05) (Fig. 4a). Similarly, rapamycin and 3-MA respectively increased and decreased the number of autophagosomes (Fig. 4b) and LC3+ cells (Fig. 4c). Furthermore, autophagy induction by rapamycin significantly decreased the in situ expression of COL2 and AGG in the MSC pellets, whereas 3-MA-mediated inhibition of autophagy had the opposite effects (Fig. 5a). Consistent with this, COL2, AGG, and SOX9 mRNA levels were respectively decreased (*p* < 0.05) and increased (*p* < 0.05) after rapamycin and 3-MA treatment (Fig. 5b–d). Taken together, autophagy has an inhibitory effect on MSC chondrocyte differentiation.

### LIPUS stimulates the chondrogenic differentiation of MSCs by inhibiting autophagy

Consistent with results presented before, LIPUS stimulation significantly decreased autophagy in the differentiating MSCs compared to the unstimulated cells in the presence of rapamycin (*p* < 0.05 for all indices; Fig. 4a–c). However, compared to the 3-MA-treated cells, the degree of autophagy was still significantly higher in the rapamycin-treated cells, even after LIPUS stimulation (Fig. 4a–c). In addition, compared to the unstimulated state, LIPUS stimulation also increased the chondrogenic markers in the differentiating MSCs in the presence of rapamycin. However, LIPUS-mediated chondrogenesis was more pronounced following additional autophagy inhibition by 3-MA compared to that after rapamycin treatment (Fig. 5a-d). Taken together, LIPUS can enhance MSC differentiation into chondrocytes by inhibiting autophagy, although it cannot completely block the autophagic pathway.

## Discussion

The aim of this study was to determine the role of autophagy in chondrogenesis of MSCs and whether LIPUS affects chondrogenesis of MSCs via regulation of autophagy. We found that autophagy activation suppressed chondrogenesis of MSCs, but autophagy inhibition promoted chondrogenesis of MSCs. In addition, we demonstrated that LIPUS promoted chondrogenesis of MSCs via autophagy inhibition.

The lack of regenerative ability in chondrocytes and the absence of nerves and vasculature in cartilage severely limits articular cartilage repair after injury. Autologous chondrocyte implantation (ACI) is a promising therapeutic option for severe cartilage defects and has shown satisfactory clinical outcome [27–29]. However, the outcome of ACI is limited by insufficient number of chondrocytes, the lack of specific niches within the articular cartilage, and individual variability [30–32]. MSCs can proliferate extensively ex vivo while maintaining their multipotent differentiation abilities, making them an ideal cell type for cell-based repair strategies [33, 34]. In addition, BMSCs are easily isolated and can differentiate into various lineages, including the chondrocytes, under optimal conditions [35].

The chondrogenic differentiation of MSCs is affected by soluble biological factors, cell-cell interactions, and the local microenvironment [36]. Several studies have demonstrated in vitro chondrogenesis of MSCs in pellet culture or on three-dimensional scaffolds in the presence of TGF-β, accompanied by the synthesis of cartilage-specific matrix proteins [37–41]. Consistent with these reports, we found that the chondrogenesis-related genes COL2, AGG, and SOX9 were upregulated in MSCs cultured in the chondrogenic medium containing TGF-β and other soluble factors.

Autophagy, a cellular degradation process that provides energy and macromolecular building blocks, is essential for cell survival and differentiation [42] and also plays an important role in the differentiation and self-renewal of stem cells [13, 14, 43]. However, the role of autophagy in the chondrogenesis of MSCs is still poorly understood. We found that the autophagy agonist rapamycin significantly decreased the chondrogenic gene signature such as COL2, AGG, and SOX9 in the differentiating MSCs, whereas the autophagy blocker 3-MA promoted chondrogenesis, indicating an inhibitory role of autophagy in the chondrogenic differentiation of MSCs.

Mechanical stress has also been shown to be an important regulatory factor in MSC differentiation, and mechanical compression such as shear stress induces chondrogenesis in MSCs by upregulating the chondrogenic genes [44, 45]. LIPUS also produces mechanical stress in the form of acoustic waves and enhances the matrix gene expression in mature chondrocytes [17, 19, 46]. Furthermore, our and others’ studies have shown that LIPUS augmented TGF-β1-induced chondrogenic differentiation of MSCs [20, 21]. Ebisawa.et al. indicated that pellet culture of MSCs is essential for the induction of chondrocyte differentiation and that TGF both accelerates differentiation and facilitates acquisition of cell machinery to respond to the LIPUS signal [20]. In our previous study, we also indicated that LIPUS promoted TGF-β1-induced chondrogenesis of MSCs, represented by increased expression of COL2, AGG, and SOX9 genes. In addition, as integrins are a type of transmembrane cell surface stress receptor and mechanistic target of the rapamycin (mTOR) plays a key role in autophagy, we found that LIPUS increased the integrin and p-mTOR expression of MSCs. Moreover, the positive effects of LIPUS on chondrogenesis of MSCs were prevented by integrin and mTOR inhibitors. These results suggested us that integrin-mTOR signaling pathway might mediate the LIPUS-induced inhibition of autophagy [21]. In the present study, we found that LIPUS enhanced chondrogenesis by inhibiting autophagy. However, addition of an autophagy agonist (rapamycin) suppressed the pro-chondrogenic effects of LIPUS to some extent, indicating the involvement of other pathways in MSC chondrogenesis. Nevertheless, our findings provide new insights into cell-based articular cartilage regeneration.

## Conclusion

In conclusion, autophagy plays a key role in chondrogenesis of MSCs, and LIPUS promotes the chondrogenic differentiation of MSCs via autophagy inhibition. Therefore, we deduce that autophagy plays a key role in the promotional effects of LIPUS on the chondrogenesis of MSCs. Our findings provide the mechanistic basis for cartilage repair using LIPUS and MSCs.

## Abbreviations

3-MA: 3-Methyladenine
ACI: Autologous chondrocyte implantation
AGG: Aggrecan
ANOVA: Analysis of variance
BMSCs: Bone marrow-derived mesenchymal stem cells
BSA: Bovine serum albumin
COL2: Type II collagen
DAB: Diaminobenzidine
DMEM: Dulbecco’s modified Eagle’s medium
DNA: Deoxyribonucleic acid
ECM: Extracellular matrix
FBS: Fetal bovine serum
FITC: Fluorescein isothiocyanate
GAPDH: Glyceraldehyde 3-phosphate dehydrogenase
IgG: Immunoglobulin G
LIPUS: Low-intensity pulsed ultrasound
MSCs: Mesenchymal stem cells
mTOR: Mechanistic target of the rapamycin
PBS: Phosphate-buffered saline
RNA: Ribonucleic acid
SD: Sprague-Dawley
SD: Standard deviation
SDS-PA: Sodium-dodecyl sulfate polyacrylamide
SOX9: SRY-related high mobility group-box gene 9
TGF: Transforming growth factor
